# Hallux gangrene following first metatarsophalangeal arthrodesis

**DOI:** 10.1093/jscr/rjaf1060

**Published:** 2026-01-08

**Authors:** Nicholas Frappa, Danil Chernov, Morgan Dillon, Matthew G Alben, Josh Slowinski, Gabrielle Hartman, Jennifer Gurske-dePerio

**Affiliations:** Department of Orthopaedics, Jacobs School of Medicine and Biomedical Sciences, 955 Main Street, Buffalo, NY 14203, United States; Department of Orthopaedics, Jacobs School of Medicine and Biomedical Sciences, 955 Main Street, Buffalo, NY 14203, United States; Department of Orthopaedics, Jacobs School of Medicine and Biomedical Sciences, 955 Main Street, Buffalo, NY 14203, United States; Department of Orthopaedics and Sports Medicine, University at Buffalo, 462 Grider Street, Buffalo, NY 14215, United States; Department of Orthopaedics and Sports Medicine, University at Buffalo, 462 Grider Street, Buffalo, NY 14215, United States; Department of Orthopaedics and Sports Medicine, University at Buffalo, 462 Grider Street, Buffalo, NY 14215, United States; Department of Orthopaedics and Sports Medicine, University at Buffalo, 462 Grider Street, Buffalo, NY 14215, United States

**Keywords:** hallux rigidus, first metatarsophalangeal arthrodesis, postoperative gangrene, microvascular ischemia, digital ischaemia

## Abstract

Hallux rigidus is commonly treated with first metatarsophalangeal (MTP) arthrodesis, a procedure with high success and low complication rates. We present a rare case of postoperative hallux gangrene following first MTP arthrodesis in a 44-year-old woman with celiac disease and no conventional vascular risk factors. Despite normal dorsalis pedis and posterior tibial pulses, the great toe developed ischemic changes on postoperative Day 1, progressing to dry gangrene requiring amputation at the MTP joint. Angiography revealed no proximal obstruction, supporting a microvascular etiology. Potential mechanisms include vasospasm, small-vessel thrombosis or autoimmune-mediated vasculitis. This case underscores the importance of early recognition of digital ischemia after forefoot surgery, even in healthy patients, and highlights the possible role of autoimmune disease in postoperative microvascular compromise.

## Introduction

Hallux rigidus, defined as degenerative arthritis of the first metatarsophalangeal (MTP) joint, is a common forefoot pathology that impairs gait and quality of life [1, 2]. When conservative measures fail, first MTP joint arthrodesis is widely regarded as the gold-standard surgical treatment [1, 2]. This procedure reliably alleviates pain and corrects deformity, with fusion rates exceeding 90% and most patients reporting high satisfaction [1, 3].

Although generally safe and effective, complications can occur. The most frequent include nonunion, malunion, hardware irritation, infection, and delayed wound healing [4, 5]. Vascular compromise is exceedingly rare, and to our knowledge no prior cases of postoperative gangrene have been reported after first MTP arthrodesis. Only isolated reports exist following other forefoot procedures, including hallux gangrene after revision hallux valgus correction, digital necrosis after second-toe arthrodesis complicated by cryoglobulinemia, and ischemia from iatrogenic arterial injury in adolescent hallux valgus surgery [6–8].

We present a rare case of hallux gangrene following first MTP arthrodesis in a healthy woman with celiac disease. While the precise mechanism is uncertain, the case illustrates that ischemia can occur despite normal vascular examinations and highlights the importance of considering atypical etiologies when perfusion deficits develop postoperatively. The patient provided consent to submit case data for publication.

## Case presentation

**Figure 1:**
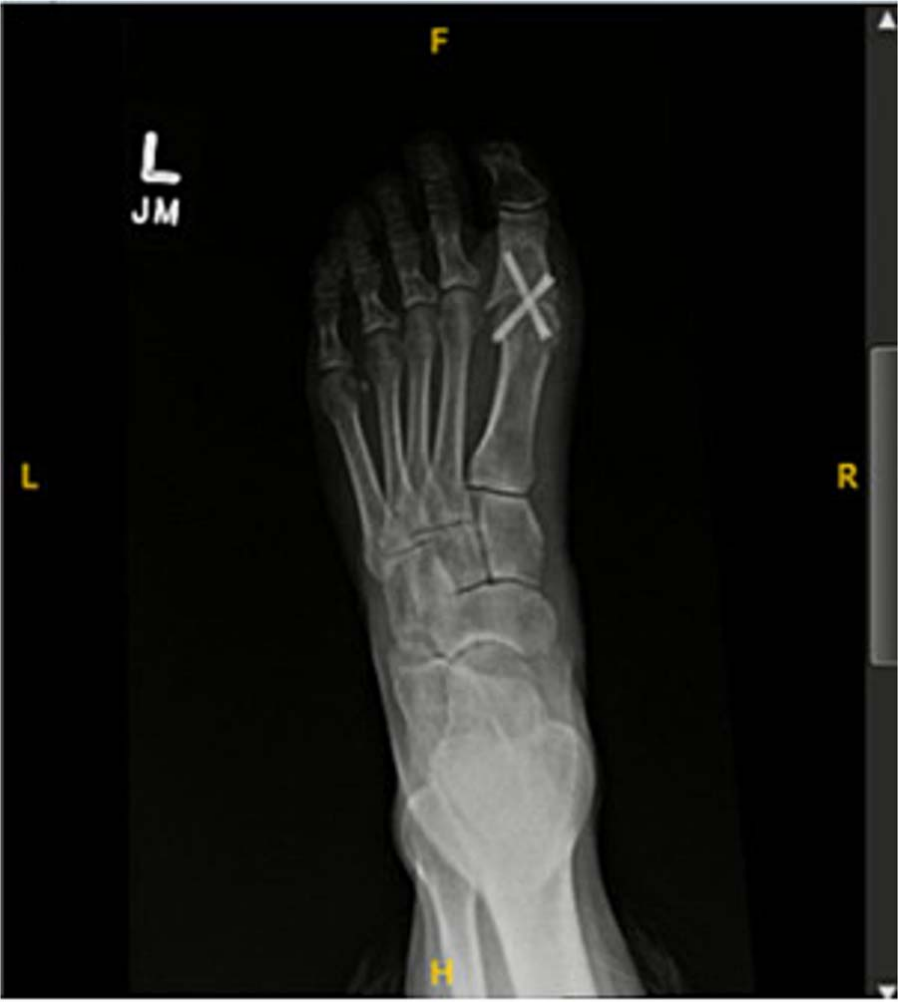
Postoperative radiograph showing first metatarsophalangeal joint arthrodesis with two headless compression screws.

A 44-year-old active woman, with a body mass index of 19.7, presented with symptomatic left hallux rigidus. Her past medical history included only celiac disease; she had no history of smoking, diabetes, peripheral vascular disease, or other conventional vascular risk factors. Her sole medication was spironolactone. She underwent standard first MTP arthrodesis with articular surface preparation, demineralized bone matrix, and fixation using two headless compression screws (Fig. 1). A tourniquet was applied just above the ankle; however, the exact duration was not available because the procedure was performed at an outside hospital. She received perioperative cefazolin prophylaxis.

**Figure 2:**
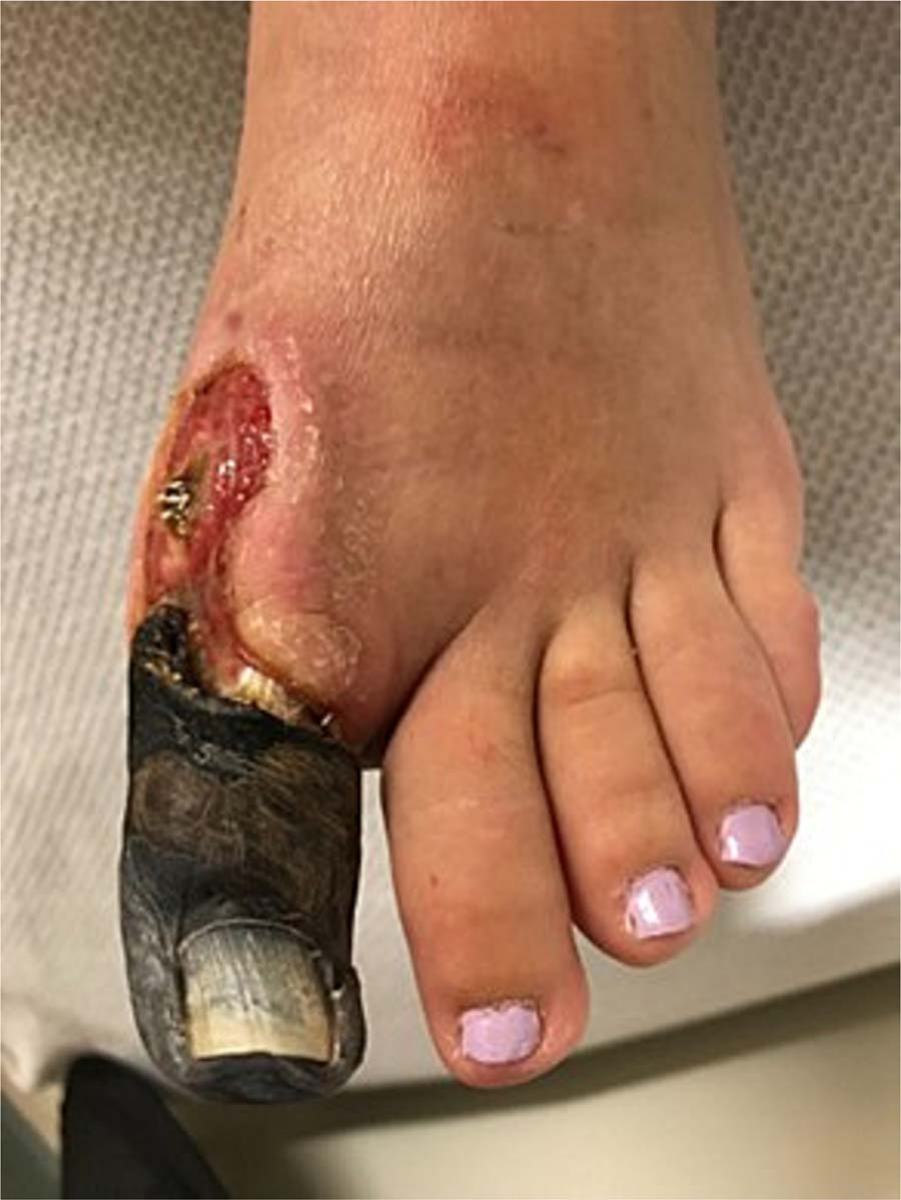
Clinical photograph showing dry gangrene of the hallux.

On postoperative day 1, the great toe became blue and painful. Vascular consultation was obtained. Angiography demonstrated impaired distal perfusion without a correctable lesion. Despite wound care, debridement, hyperbaric oxygen therapy, and oral cephalexin, the toe progressed to dry gangrene involving the entire hallux (Fig. 2). On examination, gangrene extended to the MTP joint with surrounding hyperemia but no erythema. The hallux lacked capillary refill and sensation, yet dorsalis pedis and posterior tibial pulses were 2+ (normal amplitude). Sensation and motor strength in the lesser toes were intact.

**Figure 3:**
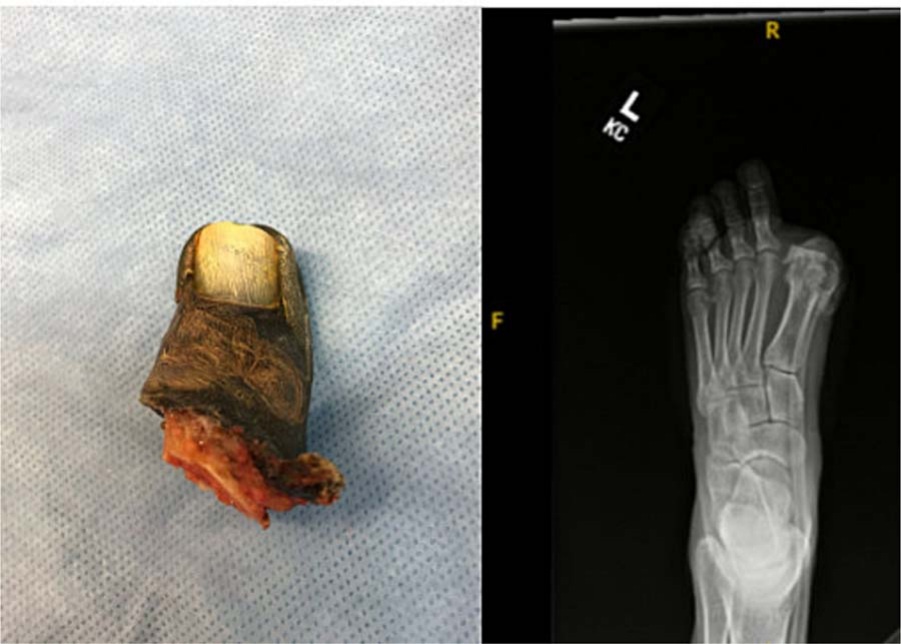
Gross specimen of amputated hallux (left) and postoperative radiograph following metatarsophalangeal joint amputation and screw removal.

**Figure 4:**
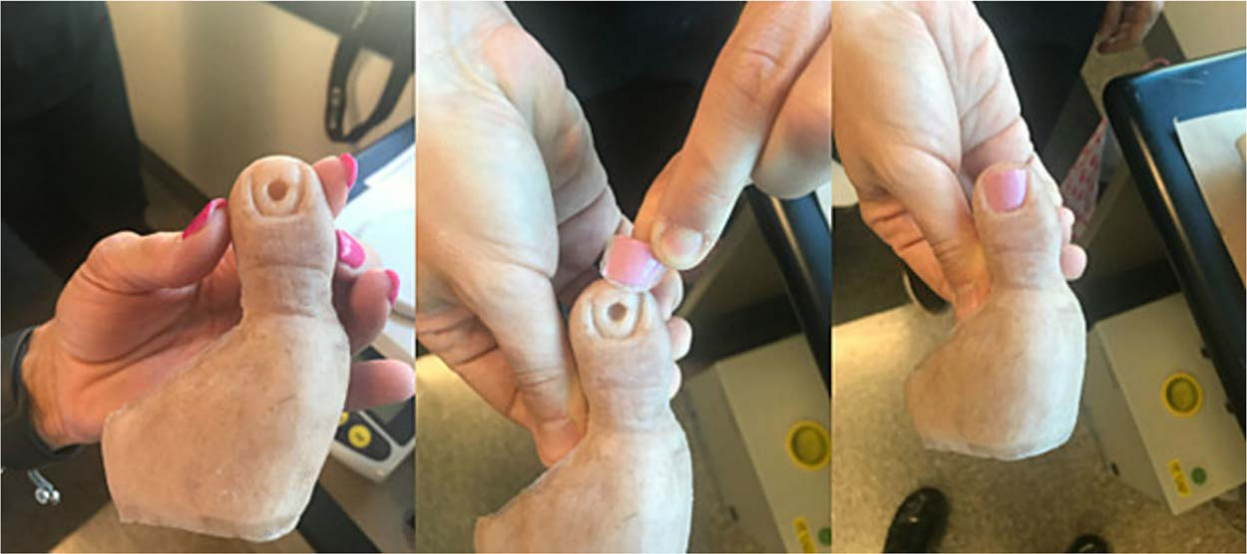
Custom prosthetic device for the great toe.

**Figure 5:**
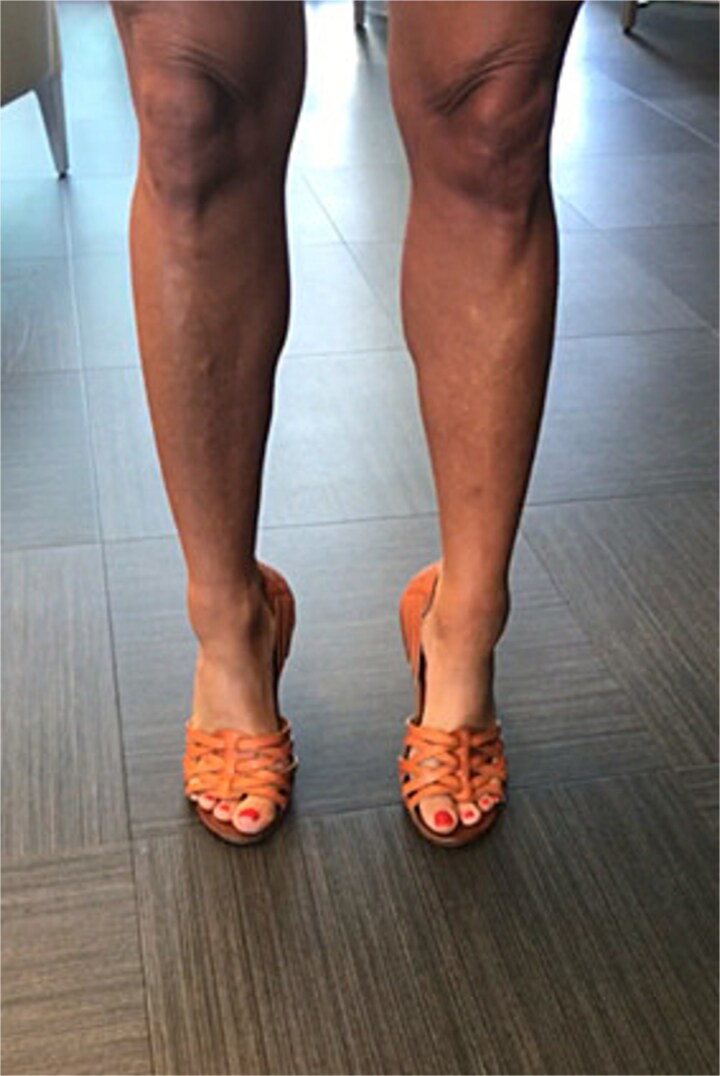
Patient wearing custom great toe prosthesis with restored cosmesis and function.

**Figure 6:**
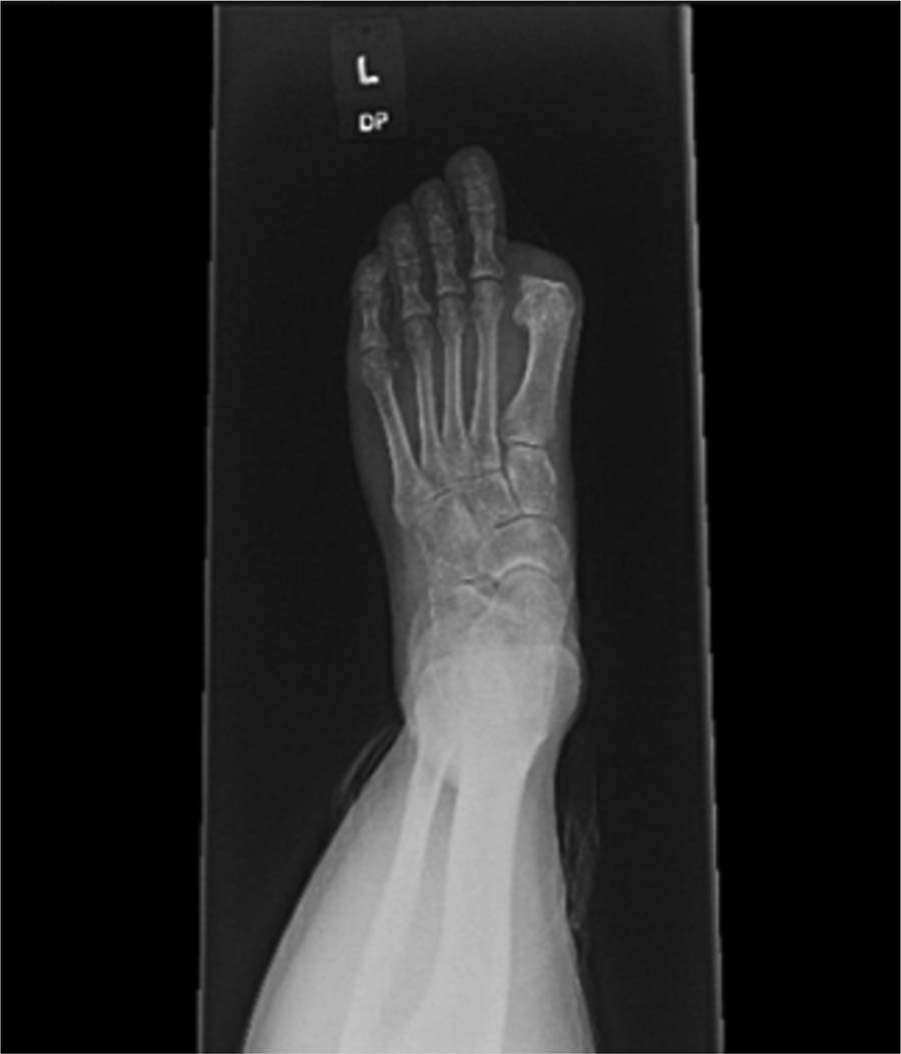
Radiographs at one year demonstrating satisfactory healing.

At 8 weeks, she underwent left hallux amputation at the MTP joint with removal of both screws, resection of nonviable tissue, and application of negative pressure wound therapy (NPWT) (Fig. 3). Intraoperative cultures demonstrated polymicrobial growth, including *Pseudomonas aeruginosa* and *Staphylococcus aureus*. She was treated with oral trimethoprim–sulfamethoxazole based on susceptibilities and infectious disease recommendations, with continued NPWT. The wound healed uneventfully, and she returned to full activity. Following wound healing, she was fitted with a custom great toe prosthetic device (Fig. 4). At 1 year, she reported no pain and had resumed gym workouts without limitation. Clinical examination showed good prosthetic function (Fig. 5), and radiographs demonstrated satisfactory healing (Fig. 6).

## Discussion

To our knowledge, postoperative gangrene has not been reported after first MTP arthrodesis for hallux rigidus. Similar cases of digital ischemia have been described following other forefoot procedures, all characterized by preserved pulses and absence of large-vessel occlusion—findings that point toward a microvascular cause [6–8].

Several mechanisms may underlie such events. One possibility is direct arterial trauma or thrombosis at the digital level from surgical manipulation [8]. Vasospastic phenomena represent another explanation; operative stress, pain, or even cold exposure can provoke prolonged vasospasm resembling Raynaud’s phenomenon in susceptible individuals [9]. A third consideration is small-vessel occlusion related to hypercoagulability or embolic events, which may occur postoperatively even in the absence of a known coagulopathy [10, 11]. Finally, an autoimmune-mediated process must be considered. Our patient’s celiac disease is noteworthy in this regard, as it has been linked to other autoimmune disorders and, rarely, systemic vasculitis [12, 13]. Together, these possibilities suggest that a latent vasospastic or autoimmune tendency could have precipitated ischemia in this case.

In our case, angiography showed no proximal obstruction, and ischemia was recognized only after gangrene had developed, necessitating amputation. This mirrors prior reports, where most patients required partial or complete toe amputation despite supportive therapies. While hyperbaric oxygen, vasodilators, or immunosuppression may help if initiated early, once dry gangrene occurs, surgical resection is typically required [7, 14]. This case highlights the importance of early recognition of ischemia after forefoot surgery, even in healthy patients. Prompt vascular and rheumatologic evaluation should be considered, particularly when autoimmune disease is present. Awareness of rare microvascular complications may allow earlier intervention and limb salvage.

## Conclusion

This case highlights a rare but serious complication of first MTP arthrodesis. Even in healthy patients with normal vascular exams, unexplained postoperative ischemia warrants urgent evaluation. Multidisciplinary involvement, particularly with vascular and rheumatologic specialists, may help identify vasospastic or autoimmune contributors. Early recognition and intervention are critical for limb salvage, and clinicians should remain aware that autoimmune conditions such as celiac disease may confer unique microvascular risks.
